# Efficacy and Safety of Triple Monoamine Reuptake Inhibitors for Major Depressive Disorder: Meta-analysis of Randomised Controlled Trials

**DOI:** 10.1192/j.eurpsy.2026.10424

**Published:** 2026-07-31

**Authors:** Z. Kahveci, D. Karaçam

**Affiliations:** Psychiaty, Istanbul University-Cerrahpşa, Istanbul, Türkiye

## Abstract

**Introduction:**

Major depressive disorder (MDD) is a leading cause of disability, and current antidepressants show limited efficacy and tolerability. Triple reuptake inhibitors (TRIs; serotonin–norepinephrine–dopamine) may improve outcomes, particularly anhedonia. Several TRIs (toludesvenlafaxine, amitifadine, liafensine, GSK372475) have been tested in randomized controlled trials (RCTs), but results remain uncertain.

**Objectives:**

To evaluate the efficacy and safety of TRIs versus placebo in adults with MDD.

**Methods:**

Systematic searches of PubMed, Embase, and CENTRAL were conducted (Figure 1). Eligible studies were RCTs ≥6 weeks in adults with MDD, comparing TRIs to placebo/antidepressants, reporting depressive symptom scales or response/remission. Data extraction and risk-of-bias assessment were performed independently.

**Results:**

Five RCTs met criteria. Continuous outcomes (Hedges g) showed substantial heterogeneity (Figure 3). Neutral-effect agents (amitifadine, GSK372475) pooled to SMD −0.08 [−0.37, 0.22], while toludesvenlafaxine showed large effects (Mi 2022 SMD −1.03 [−1.46, −0.60]; Mi 2023 SMD −1.90 [−2.11, −1.69]); subgroup difference significant. Tolerability was generally favorable for toludesvenlafaxine; GSK372475 showed limited efficacy and more adverse events.

Binary outcome (response; RR>1 favors TRI): pooled RR = 1.36 [0.86–2.16] (Figure 2), with high heterogeneity (I²=84.5%). Benefit was driven by toludesvenlafaxine, while other TRIs showed neutral or imprecise effects.

**Image 1:**
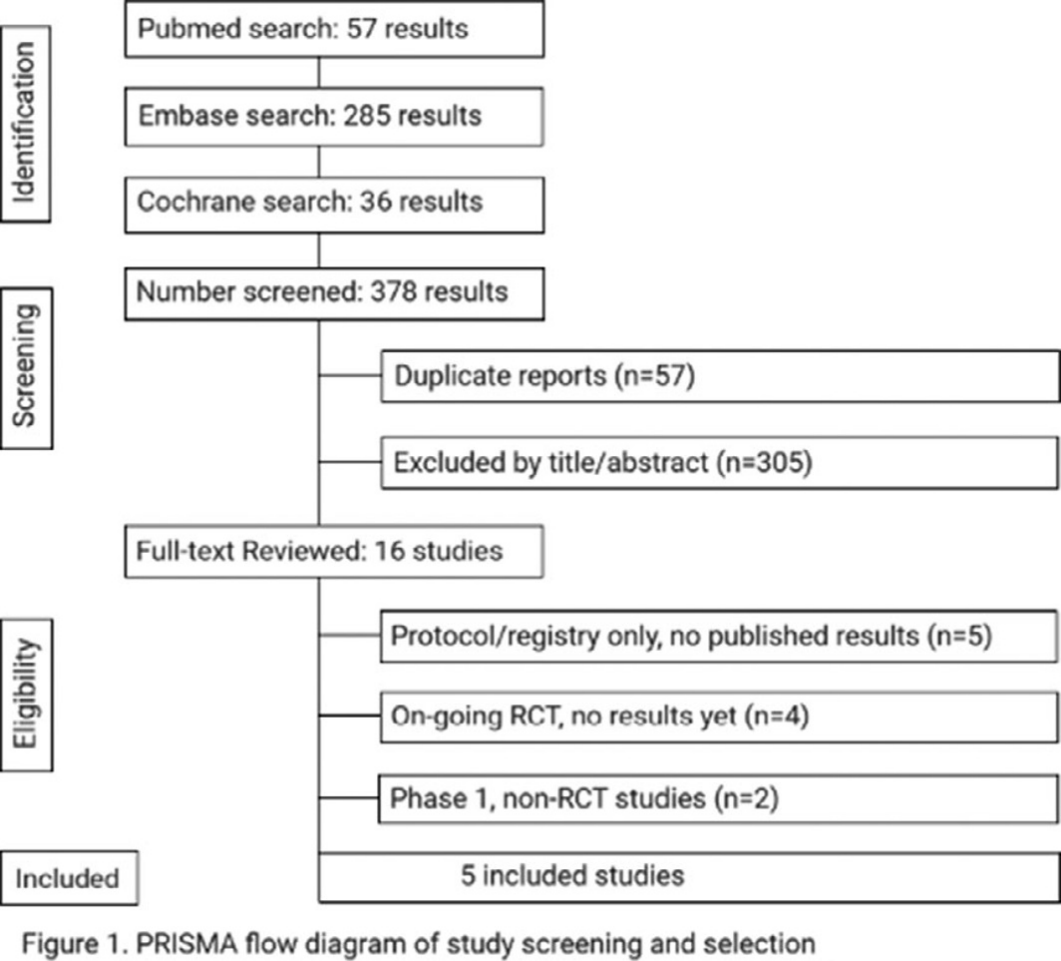
Image 1: Long description.

**Image 2:**
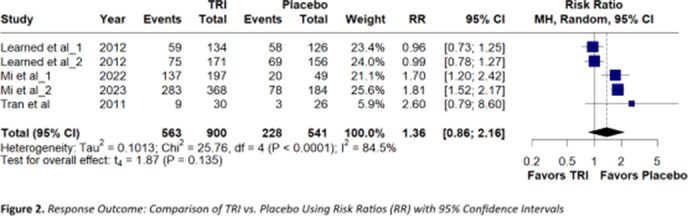
Image 2: Long description.

**Image 3:**
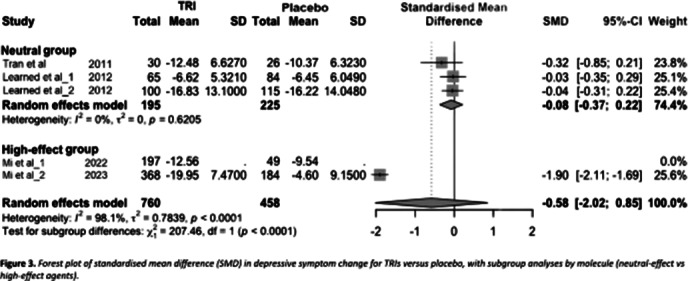
Image 3: Long description.

**Conclusions:**

Preliminary evidence suggests toludesvenlafaxine provides robust antidepressant efficacy with good tolerability, whereas amitifadine and GSK372475 show minimal benefit. Heterogeneity is molecule-driven. The meta-analysis is ongoing, and final pooled estimates will be presented at EPA 2026.

**Disclosure of Interest:**

None Declared

## Long descriptions

### Image 1: Long description

The flowchart is organized into four vertical stages labeled on the left.

1. Identification stage. Three boxes show initial search results. Pubmed search: 57 results. Embase search: 285 results. Cochrane search: 36 results.

2. Screening stage. A central box indicates Number screened: 378 results. Two horizontal branches to the right show exclusions. Duplicate reports (n=57) and Excluded by title/abstract (n=305).

3. Eligibility stage. A central box indicates Full-text Reviewed: 16 studies. Three horizontal branches to the right show further exclusions. Protocol/registry only, no published results (n=5). On-going R C T, no results yet (n=4). Phase 1, non-R C T studies (n=2).

4. Included stage. A final central box at the bottom indicates 5 included studies.

Navigate back to Image 1:.

### Image 2: Long description

A forest plot table with nine columns: Study, Year, T R I Events, T R I Total, Placebo Events, Placebo Total, Weight, R R, and 95 percent C I.

Individual study data:

* Learned et al_1 (2012): 59/134 events, 23.4 percent weight, R R 0.96 [0.73, 1.25].

* Learned et al_2 (2012): 75/171 events, 24.0 percent weight, R R 0.99 [0.78, 1.27].

* Mi et al_1 (2022): 137/197 events, 21.1 percent weight, R R 1.70 [1.20, 2.42].

* Mi et al_2 (2023): 283/368 events, 25.6 percent weight, R R 1.81 [1.52, 2.17].

* Tran et al (2011): 9/30 events, 5.9 percent weight, R R 2.60 [0.79, 8.60].

Total (95 percent C I): 563/900 T R I events, 228/541 Placebo events, 100.0 percent weight, R R 1.36 [0.86, 2.16].

Forest Plot Details:

The x-axis represents Risk Ratio on a logarithmic scale from 0.2 to 5, with a vertical line of no effect at 1. Values less than 1 favor T R I and values greater than 1 favor Placebo. The first two studies show blue squares to the left of the line, while the latter three show blue squares to the right. A black diamond at the bottom represents the pooled effect, spanning from approximately 0.86 to 2.16, crossing the line of no effect.

Heterogeneity statistics: Tau super 2 = 0.1013; Chi super 2 = 25.76, d f = 4 (P < 0.0001); I super 2 = 84.5 percent. Test for overall effect: t sub 4 = 1.87 (P = 0.135).

Navigate back to Image 2:.

### Image 3: Long description

The forest plot is divided into two main sections: Neutral group and High-effect group.

Neutral group data:

* Tran et al 2011: T R I (Total 30, Mean -12.48, S D 6.6270), Placebo (Total 26, Mean -10.37, S D 6.3230), S M D -0.32, 95% C I [-0.85, 0.21], Weight 23.8%.

* Learned et al_1 2012: T R I (Total 65, Mean -6.62, S D 5.3210), Placebo (Total 84, Mean -6.45, S D 6.0490), S M D -0.03, 95% C I [-0.35, 0.29], Weight 25.1%.

* Learned et al_2 2012: T R I (Total 100, Mean -16.83, S D 13.1000), Placebo (Total 115, Mean -16.22, S D 14.0480), S M D -0.04, 95% C I [-0.31, 0.22], Weight 25.4%.

* Random effects model: Total T R I 195, Total Placebo 225, S M D -0.08, 95% C I [-0.37, 0.22], Weight 74.4%. Heterogeneity: I super 2 = 0%, tau super 2 = 0, p = 0.6205.

High-effect group data:

* Mi et al_1 2022: T R I (Total 197, Mean -12.56), Placebo (Total 49, Mean -9.54), Weight 0.0%.

* Mi et al_2 2023: T R I (Total 368, Mean -19.95, S D 7.4700), Placebo (Total 184, Mean -4.60, S D 9.1500), S M D -1.90, 95% C I [-2.11, -1.69], Weight 25.6%.

* Random effects model: Total T R I 760, Total Placebo 458, S M D -0.58, 95% C I [-2.02, 0.85], Weight 100.0%. Heterogeneity: I super 2 = 98.1%, tau super 2 = 0.7839, p < 0.0001.

Test for subgroup differences: chi-squared sub 1 = 207.46, d f = 1 (p < 0.0001). The x-axis for the Standardised Mean Difference ranges from -2 to 2, with a vertical dashed line at 0 indicating no effect.

Navigate back to Image 3:.

